# Study of Lithium Biodistribution and Nephrotoxicity in Skin Melanoma Mice Model: The First Step towards Implementing Lithium Neutron Capture Therapy

**DOI:** 10.3390/life13020518

**Published:** 2023-02-14

**Authors:** Iuliia Taskaeva, Anna Kasatova, Dmitry Surodin, Nataliya Bgatova, Sergey Taskaev

**Affiliations:** 1 Laboratory of Ultrastructural Research, Research Institute of Clinical and Experimental Lymphology—Branch of the Institute of Cytology and Genetics, Siberian Branch of the Russian Academy of Sciences, 630060 Novosibirsk, Russia; 2 Budker Institute of Nuclear Physics, 630090 Novosibirsk, Russia

**Keywords:** skin melanoma, lithium carbonate, kidney, toxicity, lithium neutron capture therapy

## Abstract

Boron neutron capture therapy (BNCT) is one of the promising treatment methods for malignant melanoma. The main issue of this technology is the insufficient selectivity of ^10^B accumulation in tumor cells. As a result of the neutron absorption by boron, an 84% energy release occurred within the cell by the nuclear reaction ^10^B (n, α)^7^Li, which lead to tumor cell death. The use of lithium instead of boron brings a new unique opportunity—local 100% energy release—since all products of the ^6^Li (n, α)^3^H reaction have high linear energy transfer characteristics. The aim of this study was to determine the concentrations of Li in the tumor, skin, blood, brain and kidney in experimental animals with B16 melanoma and to analyze the potential Li toxicity after lithium carbonate administration at single doses of 300 and 400 mg/kg. Lithium carbonate was chosen since there is a long-term experience of its use in clinical practice for the treatment of psychiatric disorders. The inductively coupled plasma atomic emission spectrometry was used to evaluate Li concentrations in tissue samples. The accumulation efficiency of Li in the tumor was the highest at a time point of 30 min (22.4 µg/g; at a dose of 400 mg/kg). Despite the high lithium accumulation in the kidneys, the pathological changes in kidney tissues were not found. Thus, lithium may actually be used for the Li-NCT development and future studies can be conducted using ^6^Li and following irradiation of tumor cells using the schemes of lithium administration tested in this work.

## 1. Introduction

Boron neutron capture therapy (BNCT) is a type of radiation therapy and there are data demonstrating the success of this method in the treatment of various types of cancer [1,2,3]. BNCT is based on the unique high ability of the non-radioactive boron-10 (^10^B) nucleus to absorb thermal neutrons. The absorption of a neutron by boron leads to a nuclear reaction ^10^B (n, α)^7^Li with the release of energy of 2.79 MeV. In 6.1% of cases, the energy is distributed only between the lithium nucleus and the α-particle, in 93.9% of cases the lithium nucleus flies out in an excited state and emits a γ-quantum with an energy of 0.48 MeV.

The products of the nuclear reaction, the lithium nucleus and the α-particle, are characterized by a high rate of deceleration and a mean free path of these particles in water or in body tissues—5.2 and 7.5 µm, respectively—that is comparable to the cell size. The deceleration rate of the γ-quantum is significantly low; the mean free path in water is 10 cm. Consequently, the release of the main part of the energy of the nuclear reaction ^10^B (n, α)^7^Li (84%) is limited by the size of one cell. Thus, selective accumulation of boron-10 inside tumor cells and subsequent neutron irradiation should lead to the destruction of tumor cells with relatively small damage to surrounding healthy cells.

Currently, one of the main problems of successful BNCT is achieving the required levels of boron concentration in the blood and tumor cells in a certain period of time. There are three generations of boron compounds, and some of them are improving at this moment. It should be noted that the development of third-generation drugs that would fully meet these requirements is currently under investigation [2].

Taking into account the indicated problems with the selective delivery of boron to tumor cells, a radically different approach to neutron capture therapy (NCT) may be a transition to a new reaction using atoms of other elements instead of boron. For NCT to be successful, target nuclei must have a large thermal neutron absorption cross-section. Reactions that are hypothetically capable of replacing the reaction with boron should have a high neutron absorption cross section of more than 500 barns [3]. In Table 1, the reactions that were considered as an alternative to the reaction with boron are shown. However, the absorption of neutrons by most of them—^113^Cd, ^135^Xe, ^149^Sm, ^151^Eu, ^155^Gd, ^157^Gd, ^174^Hf, ^199^Hg—leads to (n, γ)-reaction, which does not provide the locality of energy release because of the low linear energy transfer characteristics of γ-rays. Locality can be provided by several (n, α)- and (n, f)-reactions due to high linear energy transfer characteristics of α-particles or nuclear fission products. However, the use of these reactions has not been studied practically because of the previously assumed high toxicity of lithium or radioactivity of uranium, plutonium and americium. These are the main reasons why, historically, the experimental work and clinical trials of neutron capture therapy are based on the use of boron.

For the successful implementation of BNCT, it is supposed that boron concentrations in the tumor should be more than 20 ppm [4]. Currently, the world’s long-term experience of the use of lithium in medicine, and the accumulated information about its toxicity, suggest the possibility of using lithium as an alternative to boron for NCT. Considering that the cross-section of the ^6^Li(n, α)^3^H reaction is four times smaller than the cross-section of the ^10^B(n, α)^7^Li reaction (940 b instead of 3835 b), and the energy released in the cell is two times higher (4.785 MeV instead of 84% from 2.79 MeV), the concentration of lithium-6 (^6^Li) in the tumor, necessary for the successful implementation of lithium neutron capture therapy (Li-NCT), according to theoretical calculations, should be 40 ppm or more. Since all products of the ^6^Li (n, α)^3^H reaction have high linear energy transfer characteristics [5] the implementation of Li-NCT provides a new unique opportunity—local 100% energy release instead of 84% [6]—as in the case of BNCT. The mechanisms of lithium entering the tumor cells are not yet fully understood; however, it is known that lithium ions enter the various cells via voltage-gated sodium channels since their permeability is the same for both Na^+^ and Li^+^ ions [7,8]. The efflux of lithium from the cell to the extracellular space occurs mainly due to the electrically neutral Na^+^—Li^+^ pump [8]. Thus, Li-NCT may represent a technique for the potentially selective elimination of tumor cells.

When the concentration of lithium-6 in tumor cells is 40 ppm, the contribution of the dose of the nuclear reaction of neutron absorption by lithium will be 55% of the total physical dose, which is sufficient for therapy with a selective accumulation of lithium in tumor cells (in the calculation, we used data presented in Figure 12 in the work [9]). Of course, a higher concentration of lithium-6 in tumor cells will only improve the quality of therapy.

Lithium enriched with the isotope lithium-6 should be used for therapy. The degree of enrichment can be very high—about 99%. Another stable isotope of lithium is lithium-7. If ^6^Li is characterized by a large thermal neutron capture cross-section (940 b), then ^7^Li is characterized by a very small neutron capture cross-section (45 mb). The capture of a neutron by lithium-7 leads to the appearance of the lithium-8 isotope, which almost instantly turns into the beryllium-8 isotope as a result of beta minus decay. The latter also decays almost instantly into two α-particles with a total energy of 17.34 MeV [10]. Therefore, the capture of a neutron by lithium-7, as well as by lithium-6, provides a local 100% energy release even 3.6 times greater. However, lithium-7 is 5.700 times less effective for therapy than lithium-6 since the neutron capture cross-section is 20.900 times smaller. Since the presence of lithium-7 does not lead to negative consequences, the enrichment of lithium in the lithium-6 isotope is not required to be extremely high and 99% enrichment seems quite reasonable.

Note that one of the reaction products, tritium, is radioactive. Tritium has a half-life of 12.32 years and its decay releases 18.6 keV of energy. The electron’s kinetic energy varies with an average of 5.7 keV, while the remaining energy is carried off by the nearly undetectable electron antineutrino. Since the energy of the electron is almost three orders of magnitude less than the energy of the ^6^Li (n, α)^3^H reaction, the accumulation of tritium should not pose a radiation hazard.

Considering all the above, the use of lithium as an analogue of boron for conducting NCT appears to be a promising strategy for the further development of NCT. Thus, the aim of this study was to determine the concentrations of lithium in the tumor, surrounding tissues, and organs distant from tumor growth in experimental animals with B16 melanoma, as well as to analyze the potential toxicity of lithium in the dosages used to assess the possibility of using lithium in NCT.

## 2. Materials and Methods

### 2.1. Reagents

Lithium carbonate (Li_2_CO_3_) was obtained from the “Novosibirsk Rare Metals Plant” (Siberia, Russia). Osmium tetroxide (OsO_4_) was obtained from Sigma-Aldrich Corp. (St. Louis, MO, USA) and Epon was obtained from Serva (Heidelberg, Germany). In this work, lithium carbonate was chosen since there is a long-term experience of its use in clinical practice, in particular for the treatment of psychiatric diseases [11,12,13,14].

### 2.2. Cell Lines

The B16 mouse melanoma cell line was obtained from the Institute of Cytology and Genetics, Siberian Branch of the Russian Academy of Sciences, Novosibirsk, Russia. The SK-Mel-28 human melanoma cell line and the BJ human fibroblasts were obtained from the ‘Center for Genetic Resources of Laboratory Animals’ of the Institute of Cytology and Genetics, Siberian Branch of the Russian Academy of Sciences, Novosibirsk, Russia. The cells were cultured in DMEM/F12 medium (Biolot, Saint Petersburg, Russia) supplemented with 10% fetal bovine serum (HyClone, Logan, UT, USA) and gentamicin 50 μg/mL (Dalchimpharm, Khabarovsk, Russia) in 75 cm^2^ culture flasks (Thermo Fisher Scientific, Waltham, MA, USA). Cells were maintained at 37 °C in a humidified atmosphere of 5% CO_2_ in the air. Cell lines were subcultured using a trypsin-EDTA solution 2–3 times a week in a ratio of 1:3–1:5. All the cells were involved in the experiment at the 3rd passage.

### 2.3. MTT-Test

Cells were seeded in 96-well plates at a density of 4 × 10^4^ cells per well in a complete medium for 24 h. Then cells were incubated for 24 h at 37 °C with lithium carbonate (LC) at lithium concentrations ranging from 10 to 640 µg/mL. At the end of the incubation period, the medium was replaced and 10 µL of 5 mg/mL MTT (3-(4,5-dimethylthiazol-2-yl)-2,5-diphenyltetrazolium bromide) was added to each well. The plates were incubated for 4 h, and then cells were treated with 100 µL of DMSO (dimethyl sulfoxide, Biolot, Russia) for 10 min at 37 °C. Absorbance at 595 nm was measured with a microplate reader Multiskan SkyHigh (Thermo Fisher Scientific, Waltham, MA, USA). Data were expressed as a percentage of the viability of control.

### 2.4. In Vivo Studies

**Figure 1:**
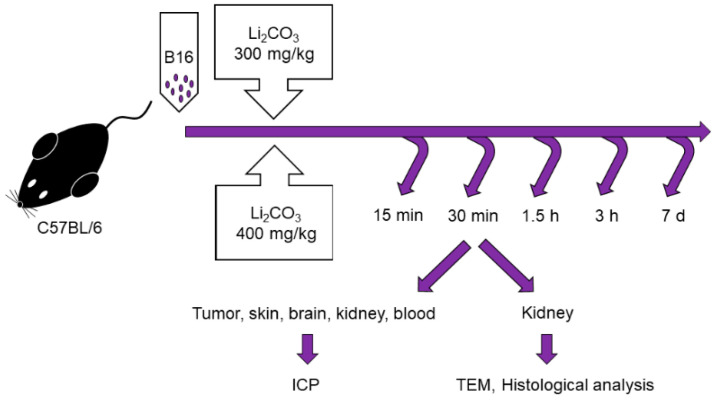
Experimental design for the assessment of lithium biodistribution in melanoma, skin, brain, kidney and blood, as well as for the evaluation of acute kidney injury in animals treated with a single dose of lithium carbonate (Li_2_CO_3_, 300 mg/kg or 400 mg/kg) *per os*. ICP—inductively coupled plasma atomic emission spectrometry, TEM—transmission electron microscopy.

Male C57BL/6 mice of 10–12 weeks of age, with weights of 20–22 g were obtained from the Institute of Cytology and Genetics, Siberian Branch, Russian Academy of Sciences, Novosibirsk, Russia. All the animals were housed in an environment with a constant room temperature (23 °C), natural day/night light cycle, and standard laboratory food and water were provided. Mice were randomly divided into eleven experimental groups (*n* = 5/group: control group with intact tumor and lithium treatment groups; Figure 1). For tumor induction, cultured cells of B16 were subcutaneously injected into the right inguinal area of the mice (1 × 10^6^ cell). After tumor growth was induced, the mice were treated by administering lithium carbonate: a single dose of 300 mg/kg (Li-300 groups) or 400 mg/kg (Li-400 groups) *per os*. The volume of lithium carbonate delivery was 50 µL; it was administered at once. After 15 min, 30 min, 90 min, 180 min and 7 days of lithium administration the mice were sacrificed. The normal skin samples were obtained at a distance of 2 mm from the tumor’s surgical margin. To assess the toxic effect of LC, a comparison was carried out between the body weights of mice from experimental and control groups at all time points.

### 2.5. Lithium Biodistribution Study

Measurement of lithium (Li) concentration in tumor, skin, blood, kidney and brain was carried out by inductively coupled plasma atomic emission spectrometry (ICP AES, ICPE-9820, Shimadzu, Japan) at Budker Institute of Nuclear Physics, Novosibirsk, Russia. The device was calibrated using the Li reference standard with a mass concentration of 1 mg/mL of the Li ion (Environmental Analytical Association EAA “Eco-analytics”, Russia) in the range of 0.01–10 mg/L.

For sample preparation, 69% nitric acid HNO_3_ and 30% hydrogen peroxide H_2_O_2_ were used. Decomposition was conducted in tubes with loose lids at a temperature of 90 °C in the Dry Block Heater 2 (IKA, Staufen, Germany) until the liquid became clear. Deionized water was added to obtain a final volume of 8 mL. Samples were injected with a peristaltic pump. The final concentration of lithium was obtained using the formula: measured concentration × sample volume/organ weight.

Pharmacokinetic parameters of Li in blood, tumor, skin, kidney and brain were calculated for each set of sample data using noncompartmental analysis in PKSolver, version 2.0 [15]. The area under the curve (AUC_0-t_) and the area under the first moment curve (AUMC_0-t_) were calculated using linear trapezoidal methods. The maximum observed concentration (C_max_) and the time to maximum concentration values (T_max_) were received directly from concentration-versus-time profiles.

### 2.6. PAS-Staining

The kidney sections were stained with periodic acid-Schiff (PAS) stain and were examined for renal morphology through quantification of a glycogen-specific color using ImageJ (National Institutes of Health, Bethesda, MD, USA). A minimum of 10 fields for each kidney slide were analyzed. The images were captured with an AxioScope.A1 microscope with 40× magnification (Zeiss, Jena, Germany).

### 2.7. Transmission Electron Microscopy

The kidney specimens were fixed with a 4% paraformaldehyde and then incubated with 1% osmium tetroxide (OsO_4_) at 4 °C for 1 h. The fixed specimens were dehydrated with gradient alcohols and embedded in Epon. Next, 70–100 nm ultrathin sections were cut with a Leica EM UC 7 microtome and stained with 1% uranyl acetate and lead citrate. Electron micrographs were taken at 30.000× at 80 kV with a JEM 1400 electron microscope (JEOL, Tokyo, Japan) and analyzed using ImageJ software (National Institutes of Health, Bethesda, MD, USA). Microscopic analysis was carried out at the Multiple-access Center for Microscopy of Biological Subjects (Institute of Cytology and Genetics, Novosibirsk, Russia). The number of fenestrae of endotheliocytes of glomerular capillaries and the number of podocyte foot processes were determined for 2 μm of the glomerular basement membrane. Additionally, the thickness of the glomerular membrane and basement membrane of proximal tubular epitheliocytes, as well as the width of podocyte foot processes and slit diaphragm were measured using scale bars.

### 2.8. Statistical Analyses

Mann–Whitney nonparametric tests were used to assess differences using the statistical package Statistica 10.0 (StatSoft, Tulsa, OK, USA). Statistically significant differences were considered at *p* < 0.05. All data are reported as the mean ± SD.

## 3. Results

### 3.1. Influence of Lithium Salts on the Melanoma Cells Viability

**Figure 2:**
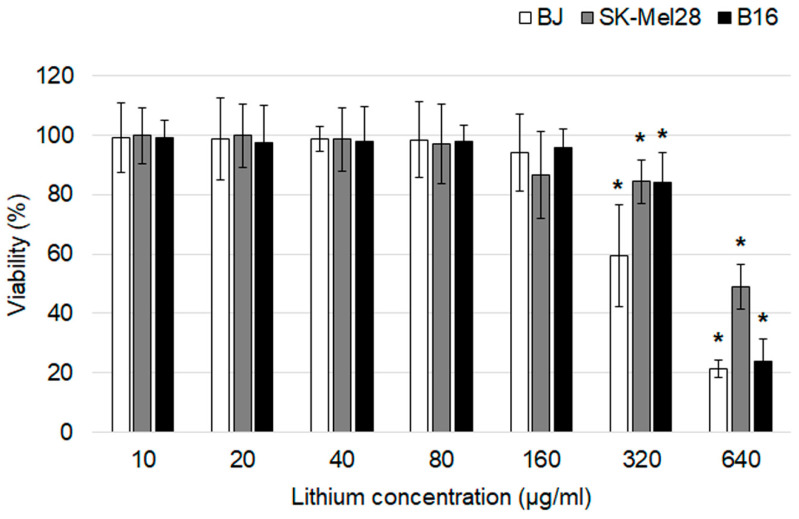
An assessment of the cytotoxic effects of lithium carbonate by an MTT assay on BJ, SK-Mel-28 and B16 cell lines (*n* = 7). * *p* < 0.05 compared with the control group. The data are presented as means ± SD.

In vitro cytotoxicity of lithium carbonate was evaluated using an MTT assay after a 24 h exposure (Figure 2). The drug didn’t affect all cell lines at the lithium concentration range of 10–160 µg/mL. Statistically significant differences in cell viability from the control groups were first obtained at the lithium concentration of 320 µg/mL. The most obvious cytotoxic effect was observed at the highest lithium concentration of 640 µg/mL for BJ and B16 cells which were characterized by significant reduction in cell viability of about 77–79%. Lithium carbonate in a lithium concentration of 640 µg/mL for SK-Mel-28 was associated with LD50.

### 3.2. Study of Lithium Accumulation in Tumor, Skin, Brain, Kidney and Blood

**Figure 3:**
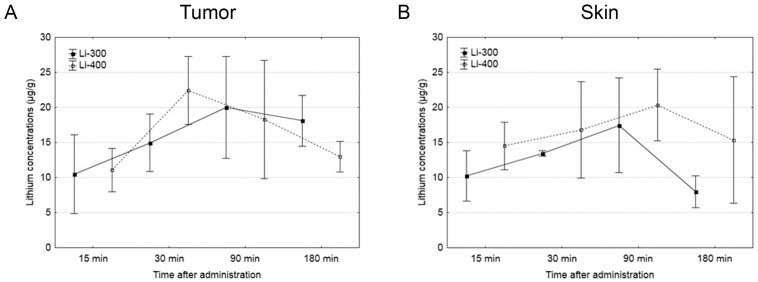

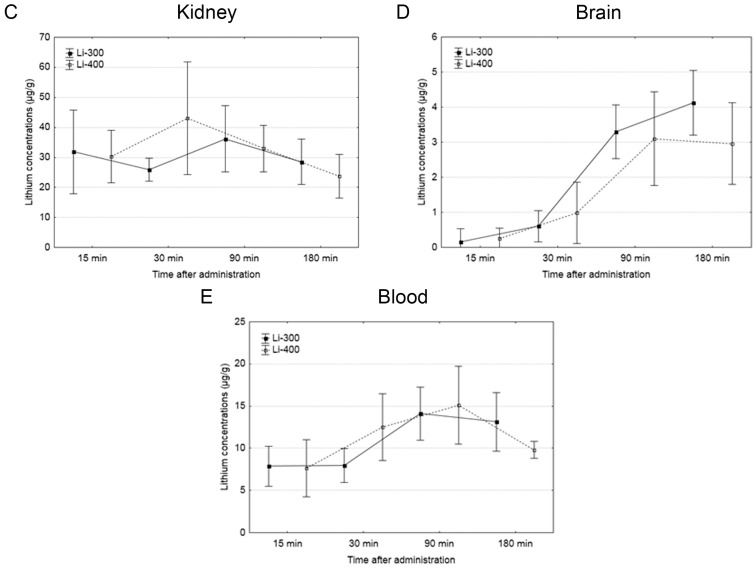
The results of the study of lithium concentration versus time profiles in (**A**) tumor, (**B**) skin, (**C**) kidney, (**D**) brain, and (**E**) blood after lithium carbonate administration at 300 mg/kg (Li-300, solid curves) and 400 mg/kg (Li-400, dashed curves) (single dose) in melanoma-bearing mice. Each point represents the mean ± SD, *n* = 5.

**Figure 4:**
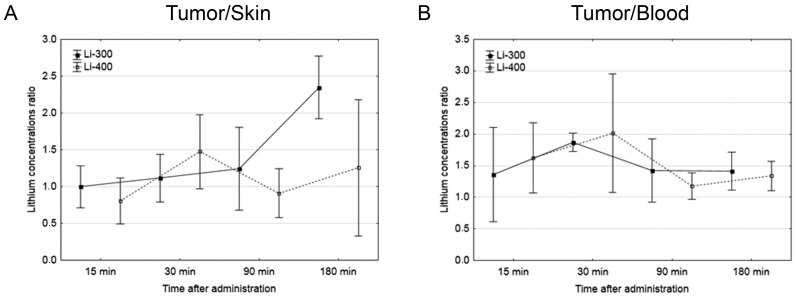
(**A**) Tumor/skin and (**B**) tumor/blood lithium concentration ratios at different time points after peroral lithium carbonate administration at 300 mg/kg (Li-300, solid curves) and 400 mg/kg (Li-400, dashed curves) (single dose) in melanoma-bearing mice. Each point represents the mean ± SD, *n* = 5.

The Li concentrations in tumor, skin, brain, kidney and blood were assessed for each of the time points after LC administration by the ICP AES method (Figure 3). The tumor/blood and tumor/skin (normal tissue) Li concentration ratios were calculated for each time point and for both drug doses (Figure 4). The accumulation efficiency of Li in the tumor was the highest at the 30 min time point in the Li-400 group (22.4 ± 4.9 µg/g). The tumor/skin ratio was 1.5 at this time point and the tumor/blood ratio was 2. For the Li-300 group, the Li concentrations ratios ranged from 1 to 2.3 (tumor/skin ratio) and from 1.4 to 1.9 (tumor/blood ratio). The same ratios for the Li-400 range from 0.8 to 1.5 and from 1.3 to 2, respectively. There were no statistically significant differences in Li accumulation between the Li-300 group and the Li-400 group. The Li concentrations in the normal brain for each dose of LC were low at all time points. The highest concentrations of Li were found in the kidneys. The experiment shows that Li concentration decreased over 7 days after a single drug administration and reached background values in all organs and tissues.

Pharmacokinetic parameters C_max_, T_max_, AUC and AUMC were obtained by analyzing the Li concentration-time curves (Table 2). The maximum concentration time (T_max_) in tumors and kidneys after LC administration at a dose of 300 mg/kg was 3 times higher than after the LC administration at a dose of 400 mg/kg, and it was 2 times higher in the brain. There were no changes between T_max_ in skin and blood after the administration of different doses of LC. There were no significant differences between the pharmacokinetic parameters AUC and AUMC in Li-300 and Li-400 groups.

### 3.3. Animal Weight Assessment

The comparison between the body weight (Table 3) of mice from experimental and control groups was carried out to evaluate the toxic effect of LC. There was a slight decrease in the body weight of mice after LC administration; however, statistically significant differences between Li-treated and control groups were not observed.

**Figure 5:**
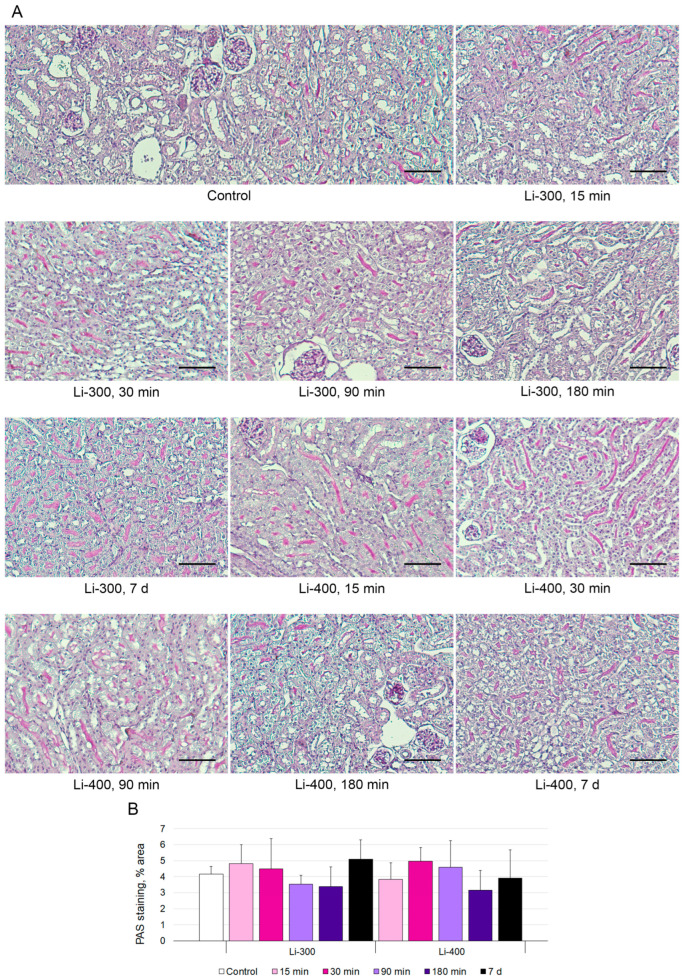
(**A**) Periodic acid–Schiff (PAS) kidney sections (*n* = 15/group) staining after 15 min, 30 min, 90 min, 180 min and 7 days of lithium carbonate administration (single dose, 300 mg/kg—Li-300 or 400 mg/kg—Li-400) *per os*. Scale bars represent 100 μm. (**B**) % area of PAS staining. The quantification of a glycogen specific color (pink) using ImageJ (National Institutes of Health, Bethesda, MD, USA) was performed to examine renal morphology. The data are presented as means ± SD. There were no statistically significant differences between the lithium-treated and control groups.

The measurements of the areas of the PAS-positive kidney tubules were performed to estimate acute kidney injury (Figure 5A). Li caused a mild decrease in staining after 180 min and 7 d of LC treatment; however, there were no statistically significant differences between Li-treated and control groups (Figure 5B).

### 3.4. Ultrastructural Organization of the Kidney Filtration Barrier and Proximal Tubules

**Figure 6:**
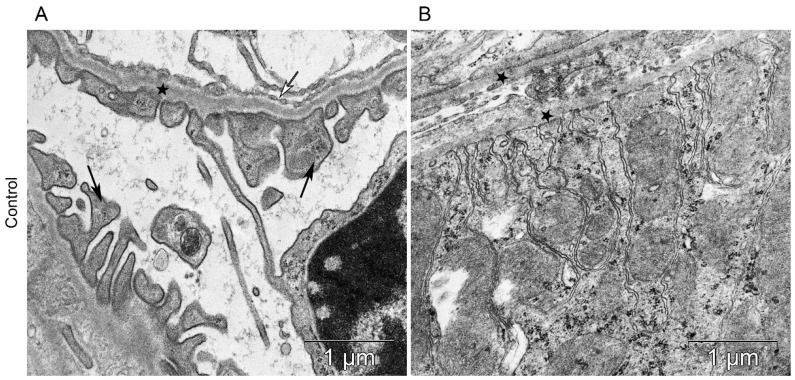
The kidney structure in conditions of distant tumor growth (control group). (**A**) Ultrastructure of the kidney filtration barrier. Glomerular basement membrane (asterisks); podocyte foot processes (black arrows); fenestrae of endotheliocytes of the glomerular capillary (white arrows). (**B**) Ultrastructure of the proximal tubule. Basement membrane of the proximal tubule (asterisks).

**Figure 7:**
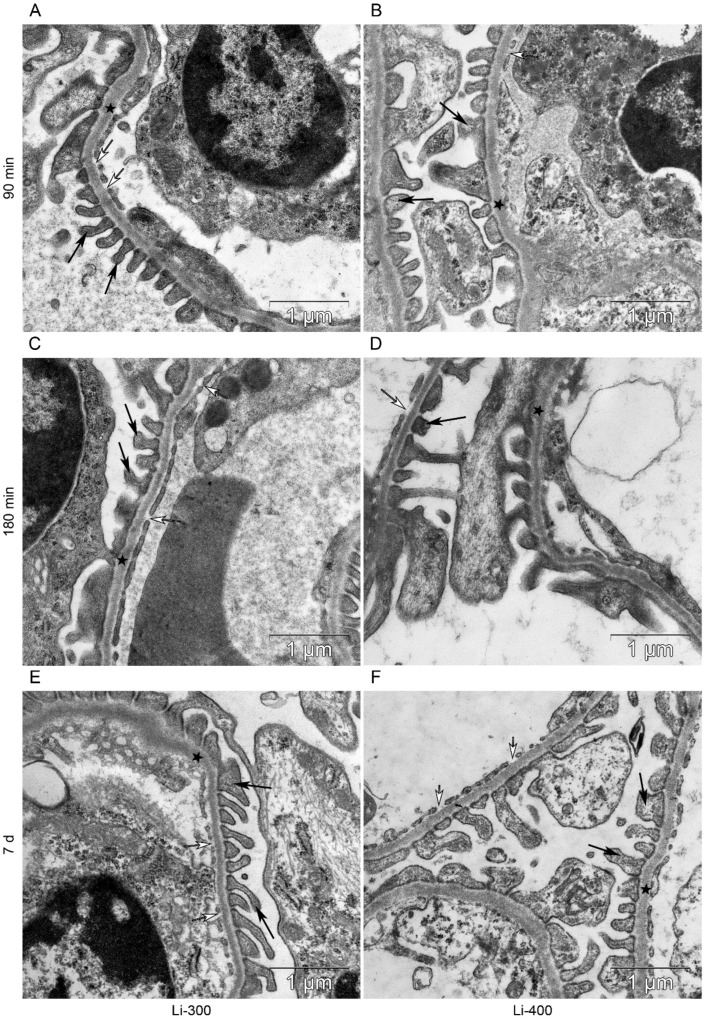
The kidney filtration barrier structure at (**A**,**B**) 90 min, (**C**,**D**) 180 min and (**E**,**F**) 7 d after peroral lithium carbonate administration at single doses of 300 (Li-300) and 400 (Li-400) mg/kg. Glomerular basement membrane (asterisks); podocyte foot processes (black arrows); fenestrae of endotheliocytes of the glomerular capillary (white arrows).

**Figure 8:**
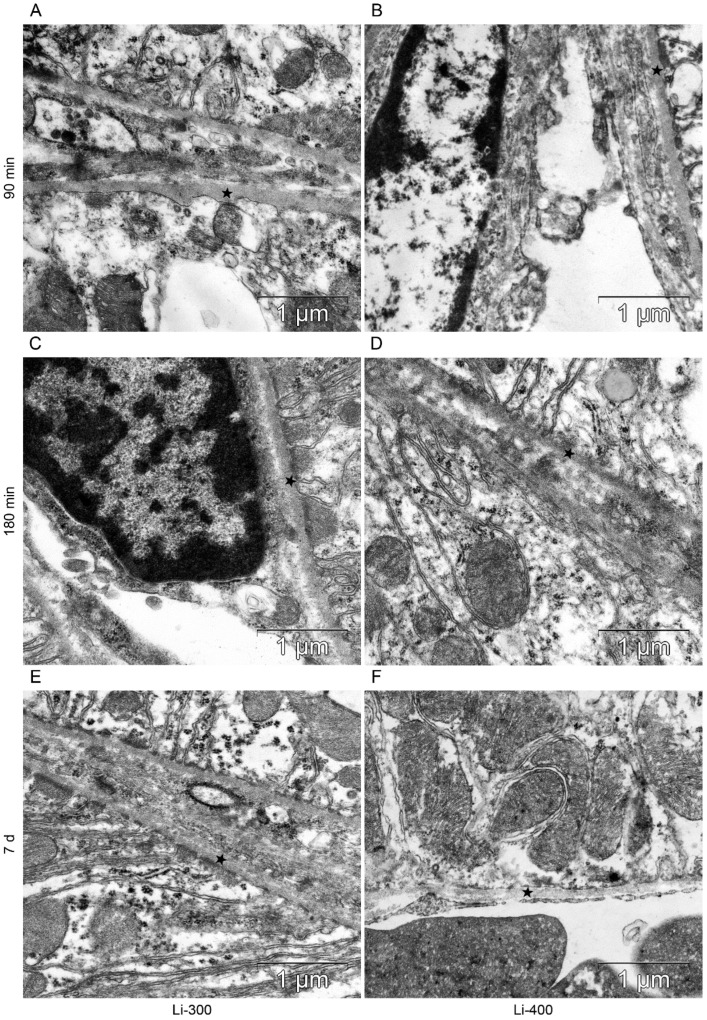
The structure of basement membrane (asterisks) of proximal tubular epitheliocytes at (**A**,**B**) 90 min, (**C**,**D**) 180 min and (**E**,**F**) 7 d after peroral lithium carbonate administration at single doses of 300 (Li-300) and 400 (Li-400) mg/kg.

All components of the mice kidney filtration barrier in the control group had a typical ultrastructural organization (Figure 6). Fenestrae of endotheliocytes of glomerular capillaries were well expressed. The heterogeneity of the ultrastructural organization of podocytes was noted. Podocyte foot processes often varied in thickness. The glomerular membrane and basement membrane of proximal tubular epitheliocytes had equable density and thickness. Lithium administration at a single dose of 300 mg/kg and 400 mg/kg did not lead to a significant change in the structure of the kidney filtration barrier (Table 4; Figure 7 and Figure 8). There were no statistically significant differences between the lithium-treated and control groups.

## 4. Discussion

### 4.1. Lithium Biodistribution: Results and Perspectives

Taking into account the importance of NCT development, ways to increase BNCT effectiveness are being actively studied all over the world. One approach is the synthesis of new boron-containing agents with a large number of boron atoms per molecule or specific compounds for each type of tumor [16]. At the moment, numerous studies are being conducted to assess the boron distribution in tumors and other organs, the final goal of which is to optimize the planned therapy. It is assumed that the elimination of tumor cells using BNCT without damage to normal cells requires a selective boron accumulation in the tumor tissue at a concentration of 15–30 μg/g of ^10^B atoms per gram of tumor tissue [17]. However, data from various studies indicate a significant heterogeneity of boron distribution in the tumor.

For example, Carpano et al. showed that boron peak concentrations in the tumor (skin melanoma) were observed 2 h after BPA administration, and ranged from 12 μg/g to 52 μg/g [16]. In addition, the authors found that the average concentration of boron in the tumor was 25.9 ± 2.6 μg/g, and the concentration ratios of tumor/blood and tumor/normal skin were 4.3 and 2.5, respectively, in 2 h after injection of 350 mg /kg BPA.

Zhang et al. investigated boron accumulation after BPA-F infusion in potential patients for BNCT: BPA-F was administered intravenously over 90 min at a dose of 350 mg/kg (two patients) and 100 mg/kg (one patient) [18]. The maximum concentration of boron was obtained immediately after the end of the BPA infusion for a dose of 350 mg/kg—after 90 min (concentrations in the tumor were in the range of 15.59–30.05 µg/g), and for a dose of 100 mg/kg—after 135 min (maximum concentration was 7.00 µg/g). The tumor/blood ratio for three patients with melanomas was in the range of 1.48–3.82, while the normal skin/blood ratio was in the range of 0.81–1.99.

Garabalino et al. studied the accumulation of boron in tumor tissues in hamsters after an intravenous infusion of BSH at a dose of 50 mg/kg [19]. The values of the maximum concentration of boron in the tumor varied from 24 to 35 μg/g, and the ratio of boron concentrations in the tumor/normal tissue varied from 1.1 to 1.8.

In this study, the peak concentration of Li in the tumor at the maximum administered dose of LC 400 mg/kg was 22 µg/g; thus, the target calculated concentration of 40 µg/g was not achieved. According to the literature data, BNCT is considered successful despite the significant variability of boron concentrations and the presence of concentrations in the tumor below the required calculated values (20 µg/g) [20,21]. In addition, the best results of BNCT studies and high concentrations of boron in the tumor, have recently been obtained by using the latest generation of boron agents, which significantly increase the selectivity of its delivery to tumor cells. These results have been obtained by using various types of liposomes [22,23], polymers [24], nanoparticles [25,26,27], mannopyranoside complexes [28] and an electrochemotherapy in combination with boron drugs [29]. Here we used LC salt, which, upon administration of Li ions, were nonselectively absorbed by tumor cells; therefore, it can be assumed that the creation of a special construction with Li based on a selective carrier, similar to boron drugs, will significantly increase the accumulation of Li in the tumor.

Furthermore, differences in boron distribution can also be observed between a single-injection mice model and the continuous-infusion mice model [30]. Thus, the route of drug administration can also have a significant impact on its distribution; in this regard, further studies may also be required to find the optimal route of Li administration. In addition, in this study, the maximum Li concentrations were observed in the kidneys, at both dosages used and at all time points, which is explained by the large participation of this organ in the elimination of lithium [31]. The kidneys also traditionally have high boron concentrations since they are the route of excretion of boron and its metabolites [16].

### 4.2. Lithium Toxicity

Depending on the symptoms caused by the Li pharmacokinetics, acute, acute-on-chronic and chronic lithium intoxication are distinguished [32]. Li is excreted predominantly by the kidneys as a free ion with great inter-individual variability and depends on factors such as age, total body weight, and renal function [33]. Body weight parameters and renal creatinine clearance have been shown to accurately predict steady-state Li concentrations by reducing inter-individual variability [34]. Accordingly, kidney damage is one of the most common and well-characterized side effects of lithium therapy, leading to the development of so-called Li-induced nephropathy. It is known that the characteristic manifestations of Li nephrotoxicity are focal nephron atrophy and interstitial fibrosis with relative preservation of glomeruli [35].

In this study, the assessment of the condition of the renal tubules and the filtration barrier of the kidney was performed. The filtration barrier of the kidney includes fenestrated endothelial cells, the glomerular basement membrane (glomerular membrane), as well as podocyte processes and slit diaphragms of podocytes [36]. The morphological study of the filtration barrier components and the PAS staining results showed that a single administration of LC at doses of 300 and 400 mg/kg does not lead to acute kidney injury.

According to the literature data, the treatment duration, age and previous episodes of Li intoxication may be the risk factors for the development of Li-induced nephropathy [37,38,39]. It is currently unknown whether the Li administration protocols will affect differences in the incidence of end-stage renal disease [35]. In line with this, it can be assumed that a single dose of lithium for the purpose of Li-NCT will not increase the risk of kidney damage, especially given the strict stratification of patients by risk factors.

The relatively safe oral administration dose of Li ranges from 450 to 1300 mg/day [33]. It is well-known that Li has a narrow therapeutic index: the target blood concentration varies from 0.5 to 1.2 mmol/L and depends, as a rule, on the excretion rate [40]. However, it was reported that the pharmacokinetics of Li in humans is significantly different from that in rodents [41].

The present study shows that the maximum concentration of Li in the blood at doses of 300 and 400 mg/kg was about 2.2 mmol/L, which exceeded the therapeutic values. Nevertheless, it is known that the half-life of Li is approximately 24 h and the obtained C_max_ of 2.2 mmol/L was recorded 90 min after drug administration, which is consistent with the data on reaching peak Li concentrations in humans in the range of 1–3 h after oral administration of the drug [42]. Registration of Li levels in human blood serum is carried out 5–7 days after the start of treatment when the equilibrium state of Li in the blood is reached [33,43]. At the same time, it is known, that moderate toxicity of Li is manifested at levels up to 2.5 mmol/L [8]. Taking the above into account, as well as the absence of significant weight loss in experimental animals and pathological changes in kidney tissues, it can be concluded that there is no acute Li toxicity in this protocol of Li administration.

Thus, this study shows for the first time the parameters of Li biodistribution in tumor tissue (skin melanoma), as well as in organs distant from tumor growth—the brain, skin, and kidneys. High concentrations of Li in tumor tissues were detected. For the first time, the tumor/blood and tumor/normal tissue (skin) concentration ratios have been determined, which will be important in the selection of the optimal NCT protocol. In conditions of tumor growth, the assessment of acute nephrotoxicity of LC was performed for the first time, and the absence of significant changes in the kidneys after administration of the drug at doses of 300 and 400 mg/kg was shown.

Currently, lithium carbonate is approved for use in clinical practice only for the treatment of psychiatric disorders, and it is not used in oncology. Future experiments should be carried out for the possible implementation of Li-NCT. Firstly, it is appropriate to determine the lithium microdistribution in the tumor, which may affect its biological effectiveness. Secondly, the experimental irradiation of tumor cells with an enriched ^6^Li drug should be carried out to assess the possibility of a lithium neutron capture reaction. Thirdly, the development and investigation of a selective lithium carrier seem to be an important direction of future Li-NCT development to improve lithium delivery to tumor cells. In this work, the oral route of drug administration was used because LC is traditionally used orally in tablet form in clinical practice [44,45]. Thus, the optimal route of LC administration should be determined in future experiments since the alternative routes of LC administration can increase drug accumulation in a tumor. Finally, the incorporation of females in the experimental study design can provide the ability to detect a sex difference in the toxicity of lithium [46].

## Figures and Tables

**Table 1:** Isotopes with high values of thermal neutron capture cross-section.

Nuclide	Prevalence, %	Half Life	Interaction	Cross-Section, b
^3^He	0.000013		(n, p)	5333
^6^Li	7.4		(n, α)	940
^10^B	20		(n, α)	3835
^113^Cd	12		(n, γ)	20,600
^135^Xe		9.14 h	(n, γ)	2,720,000
^149^Sm	14		(n, γ)	42,080
^151^Eu	48		(n, γ)	9200
^155^Gd	15		(n, γ)	61,100
^157^Gd	15		(n, γ)	259,000
^174^Hf	0.16	2 × 10^15^ years	(n, γ)	561
^199^Hg	17		(n, γ)	2150
^235^U		7 × 10^8^ years	(n, f)	681
^241^Pu		13.2 years	(n, f)	1380
^242^Am		16 h	(n, f)	8000

**Table 2:** Pharmacokinetic parameters of lithium biodistribution in tumor, skin, kidney, brain and blood after peroral administration *.

Group/Parameters	T_max_ (min)	C_max_ (µg/mL or /g)	AUC_0–180_ (min * μg/mL or /g)	AUMC_0–180_ (min * μg/mL or /g^2^)
Lithium carbonate 300 mg/kg				
Tumor	90	20.0 ± 7.2	3033.4 ± 946.5	300,882.1 ± 84,457.8
Skin	90	17.5 ± 6.8	2326.0 ± 675.5	199,903.5 ± 64,963.1
Kidney	90	32.0 ± 6.9	5029.7 ± 1098.0	481,887.0 ± 113,178.5
Brain	180	4.1 ± 1.0	458.1 ± 120.9	56,393.0 ± 13,198.9
Blood	90	14.1 ± 3.1	2067.3 ± 504.2	212,511.1 ± 52,241.0
Lithium carbonate 400 mg/kg				
Tumor	30	22.4 ± 4.9	2964.3 ± 961.4	256,485.7 ± 81,014.9
Skin	90	20.3 ± 5.1	3063.2 ± 1096.2	283,785.6 ± 115,900.1
Kidney	30	43.0 ± 18.7	5611.0 ± 1742.0	470,407.7 ± 134,435.7
Brain	90	3.1 ± 1.3	406.7 ± 190.2	46,096.8 ± 19,559.7
Blood	90	15.1 ± 4.6	2158.0 ± 593.1	197,089.6 ± 44,734.1

* The data are presented as means ± SD. There were no statistically significant differences between groups that obtained single dose lithium carbonate at 300 mg/kg or 400 mg/kg.

**Table 3:** Body weight of the mice after the administration of lithium carbonate (single dose, 300 mg/kg—Li-300 or 400 mg/kg—Li-400) *per os* *.

Group/Parameters	Body Weight, g
Control	25.8 ± 1.1
Li-300, 15 min	22.1 ± 1.9
Li-300, 30 min	22.6 ± 2.4
Li-300, 90 min	22.1 ± 2.0
Li-300, 180 min	22.7 ± 1.7
Li-300, 7 d	23.5 ± 1.5
Li-400, 15 min	22.9 ± 1.8
Li-400, 30 min	23.1 ± 1.7
Li-400, 90 min	23.2 ± 2.7
Li-400, 180 min	22.3 ± 1.0
Li-400, 7 d	23.8 ± 1.1

* The data are presented as means ± SD. There were no statistically significant differences between the lithium-treated and control groups.3.4. Kidney PAS-Staining.

**Table 4:** The electron microscopy study of kidney *.

Group/ Parameters	GBM, Thickness, μm	PFP, Width μm	PFP/ 2 µm GBM	BMPT, Thickness, μm	Fenestrae/ 2 µm GBM	Slit Diaphragm, Width, nm
Control	0.11 ± 0.01	0.3 ± 0.2	6 ± 2	0.14 ± 0.03	3.6 ± 1.7	34.6 ± 9.8
Li-300, 15 min	0.11 ± 0.01	0.3 ± 0.1	5.0 ± 1.6	0.13 ± 0.02	3.8 ± 0.8	34.8 ± 6.9
Li-300, 30 min	0.12 ± 0.01	0.3 ± 0.1	5.8 ± 1.1	0.12 ± 0.01	4.4 ± 1.5	31.1 ± 7.5
Li-300, 90 min	0.14 ± 0.02	0.3 ± 0.2	5.6 ± 2.7	0.13 ± 0.03	4.2 ± 1.7	34.4 ± 11.5
Li-300, 180 min	0.12 ± 0.02	0.3 ± 0.2	5.4 ± 0.8	0.13 ± 0.02	3.2 ± 1.3	32.4 ± 8.7
Li-300, 7 d	0.11 ± 0.01	0.2 ± 0.1	6.6 ± 2.0	0.12 ± 0.02	4.8 ± 1.3	35.5 ± 7.1
Li-400, 15 min	0.12 ± 0.01	0.2 ± 0.1	7.0 ± 0.8	0.12 ± 0.01	3.8 ± 1.1	27.1 ± 5.8
Li-400, 30 min	0.11 ± 0.02	0.2 ± 0.1	6.5 ± 1.3	0.12 ± 0.01	4.3 ± 0.5	31.1 ± 11.3
Li-400, 90 min	0.12 ± 0.02	0.3 ± 0.2	6.4 ± 1.9	0.13 ± 0.02	3.4 ± 1.6	31.1 ± 7.5
Li-400, 180 min	0.13 ± 0.02	0.3 ± 0.2	5.4 ± 2.1	0.12 ± 0.02	3.4 ± 1.5	29.1 ± 6.9
Li-400, 7 d	0.13 ± 0.02	0.2 ± 0.1	7 ± 1.5	0.13 ± 0.01	5 ± 1	35.7 ± 9.8

* The kidney morphology after 15 min, 30 min, 90 min, 180 min and 7 days of lithium carbonate administration (single dose, 300 mg/kg—Li-300 or 400 mg/kg—Li-400) *per os*. The data are presented as means ± SD. GBM—glomerular basement membrane; PFP—podocyte foot process; BMPT—basement membrane of proximal tubule; Fenestrae—fenestrae of endotheliocytes of the glomerular capillary. There were no statistically significant differences between the lithium-treated and control groups.

## Data Availability

The data presented in this study are available on request from the corresponding author. The data are not publicly available due to [the datasets are being used for other studies].
